# Rare drug allergies: Review on prevalence and test procedures 

**DOI:** 10.5414/ALX01578E

**Published:** 2017-08-04

**Authors:** R. Treudler, J.C. Simon

**Affiliations:** Klinik für Dermatologie, Venerologie und Allergologie, Universität Leipzig, Germany

**Keywords:** allergy, drug, skin test, biologics, telaprevir, hypersensitivity

## Abstract

This paper gives a review on rare hypersensitivity reactions (including allergies) to drugs. Pathogenesis, allergy tests and possible therapeutic options are discussed by presenting examples out of the following group of drugs: antiinfectious (i.e. chinolones, telaprevir), oncological (i.e. platin-based cytostatics), immunologic (i.e. cetuximab, omalizumab), others (i.e. glucocorticosteroids). Usually there is no standardized allergologcial work-up procedure. Testing must therefore take into consideration previous experiences from other authors and on general recommendations.

German version published in Allergologie, Vol. 36, No. 5/2013, pp. 210-218

## Introduction 

Which preparations may lead to adverse effects varies according to the age of the treated patients. In younger patients, antibiotics (β-lactam antibiotics in particular) are among the most frequent causative drugs, while in older patients, adverse effects are most frequently caused by NSAIDs or heparins [19]. This article gives a review of some rare drugs that can trigger allergies or hypersensitivity reactions. Rare, in many cases here, means that these drugs are only used in a relatively small portion of the total population. On the other hand, the adverse effects noted with these drugs might not at all be rare when seen with regard to the treated patient collective (e.g., HIV-infected people, cancer patients). Due to the high number of drug classes that are able to trigger adverse effects, we can only report on some selected preparations. This selection was mainly based on the newest developments with regard to anti-infectious and oncologic-immunologic drugs. 

## Pathogenesis of adverse effects 

In many cases, the underlying mechanisms of adverse drug effects have not yet been entirely elucidated. On the one hand, they can be related to the pharmacological effects of the drug, on the other hand, the adverse effects can be due to a patient’s specific hypersensitivity. For some drugs, for example, a clear association of hypersensitivity reactions with certain HLA alleles could be demonstrated. Only drug hypersensitivity reactions that are based on a well-defined immunologic mechanism are denominated as drug allergy. In this context, a clear distinction has to be made between allergic reactions and non-allergic hypersensitivity reactions where other mechanisms play a role, e.g., interference of acetylsalicylic acid (ASA) with the leukotriene system. Particularly in the case of new preparations, it is often difficult to find out whether a reaction is an allergic one or if other mechanisms (like cytokine effects, immunologic imbalances (autoimmune reactions), or cross-reactivity at receptors) are responsible for the adverse effect [27, 43]. 

As adverse effects can be associated with very heterogeneous clinical manifestations and may be based on relatively different pathogenetic factors, they are frequently classified in everyday clinical practice as immediate-type and late-type reactions [27]. From a clinical point of view, immediate-type reactions are, e.g., pruritus, urticaria, anaphylaxis; late-type reactions are exanthematous reactions with either simple (e.g., maculopapular), complex (e.g., acute generalized exanthematous pustulosis (AGEP), drug rash with eosinophilia and systemic symptoms (DRESS)), or bullous clinical pictures (e.g., Stevens-Johnson syndrome (SJS), toxic epidermal necrolysis (TEN)) [27]. 

## General considerations of the diagnosis of adverse effects 

Immediate-type reactions to drugs can in part be associated with IgE-mediated events, whereas late-type reactions can be linked to T-cell-mediated reactions. Key factors for diagnosis are positive skin and blood allergy tests. Skin tests are the most promising diagnostic test for immediate-type reactions and for late-type reactions with macular, papular, or pustular rashes (including SDRIFE, AGEP, DRESS). If, on the other hand, bullous exanthema is present, skin testing is not a promising approach [7]. 

All in all, allergologic work-up is often negative so that nonimmunological hypersensitivity has to be assumed. Incorrect test concentrations or reactions to metabolites, which cannot be assessed in current tests, can be other reasons for negative test results. General information on diagnostic work-up in drug allergy can be found in recent reviews [1, 2, 5, 7, 27, 33, 37]. In immediate-type reactions, skin prick and intradermal tests as well as examinations for specific IgE are used; in late-type reactions, a patch test and/or a late-reading intradermal test is applied. Other procedures (e.g., CAST, LTT) are less suitable for routine testing. Unfortunately, only a few preparations for standardized skin testing exist, and it is often very difficult to identify the correct test concentration (Table 1) [2, 5, 7]. Different procedures can give different results [2, 7]. In doubt, test modalities should be based upon current literature, particularly publications of allergy working groups (e.g., European Network for Drug Allergy/ENDA, Working Group Drug Allergy of the German Society for Allergology and Clinical Immunology). If necessary, (double) blind provocation testing can also be used for diagnostic work-up [1]. 

## Hypersensitivity reactions to certain drug classes 

### Anti-infectives 

**Antibiotics**

Immunologically-mediated adverse reactions have not only been shown for β-lactam antibiotics but also for several other antibiotics, these include IgE-mediated reactions against macrolides (e.g., erythromycin), aminoglycosides (e.g., streptomycin), and glycopeptides (e.g., vancomycin) [9]. Lately, quinolones have been attracting more and more attention as their increasing use has led to more frequent observations of immediate-type reactions, particularly against ciprofloxacin, but also against oflaxacin, moxifloxacin, and levofloxacin. The rate of anaphylactic reactions against quinolones has been indicated to be from 1:1.000 to 1:100.000. Positive skin prick and intradermal tests as well as the detection of specific IgE antibodies are suggestive of immunologic sensitization. Due to the irritating potential of these preparations, skin tests can lead to false-positive results and thus have to be carried out in adequate dilutions (e.g., ciprofloxacin 0.002 – 0.02 mg/mL and levofloxacin 0.025 mg/mL for intradermal testing) [10, 40]. 

**Antimycotics**

Despite its widespread use, nystatin only rarely leads to allergic reactions. Contact allergies have been described for local applications, and exanthematous reactions – sometimes also severe ones – have been seen when nystatin has been used systemically. Positive skin tests (e.g., patch test with 30% nystatin in aqueous solution or 10% in petrolatum) have been described [26, 34]. 

For terbinafine there is anecdotal evidence of symmetric drug-related intertriginous and flexural exanthema (SDRIFE) [48], pustular exanthema (AGEP or pustular psoriasis) [3, 15], and bullous exanthema (SJS or TEN) [36, 49]. Skin test are usually not helpful in the diagnostic work-up of these reactions. 

Voriconazole is a second-generation triazole that can lead to visual problems (21%) and increased liver parameters (15,6%), but also to – mainly UV-induced and probably phototoxic – exanthemas (7%) [47]. 

**Antiviral drugs**

HIV infection [14]

Abacavir hypersensitivity syndrome can occur in 5 – 8% of HIV-infected patients, typically 9 – 11 days after start of therapy. Positive patch tests suggest an immunologically-mediated reaction, and an association with HLA-B5701 has been found in Caucasians. Nowadays, adequate pharmacogenetic work-up is usually carried out before the start of therapy. 

Hypersensitivity reactions to nevirapine are observed in 13% of users, mainly in the form of a maculopapular exanthema and only in 0.5 – 1% of cases as a severe exanthema (SJS/TEN). A CD4+ T cell-dependent immune response has been postulated because the adverse effects of nevirapine are associated with HLA-DRB1*0101. Hypersensitivity reactions, some of which have been related to immunologic mechanisms, have also been described for several other preparations used in HIV therapy: for example, photoallergic reaction to efavirenz [41], exanthema in 6% of patients treated with atazanavir or > 1% of patients treated with enfuviritide [13]. 

Hepatitis [11, 30]

The proteinase inhibitor telaprevir received marketing authorization for treatment of viral hepatitis in combination with IFN + ribavirin at the end of 2011. During treatment with telaprevir, the general incidence and severity of skin rashes were increased if telaprevir was used together with peginterferon alfa and ribavirin. Skin rashes were observed in 55% of patients treated with a combination therapy of telaprevir, peginterferon alfa, and ribavirin, while 33% of patients treated with peginterferon alfa and ribavirion alone suffered from skin rashes. More than 90% of these exanthemas were of mild or moderate severity. In 0.4% of studied patients, severe drug exanthema in the form of drug rash with eosinophilia and DRESS was suspected. Less than 0.1% of patients developed SJS. Whether skin testing would be of use for the diagnostic work-up of these reactions remains unknown. 

### Oncological/immunological drugs 

Several recent oncological drugs have gained attention for their relatively typical adverse effects, which are probably based on toxic irritant effects or on biological cross-reactions rather than on immunological effects. Some examples are adverse effects to epidermal growth factor (EGFR) inhibitors (e.g., papulopustular exanthemas in up to 80% of patients, also xerosis, hypotrichosis, whitlow) or the hand-foot syndrome associated with the use of vascular endothelial growth factor (VEGF) inhibitors (sorafenib, sunitinib) [16, 43]. The following paragraphs highlight adverse effects to certain biologics and platinum-based chemotherapeutic drugs. 

**Biologics**

Immediate-type reactions have been described after administration of cetuximab (anti-EGFR), omalizumab (anti-IgE), infliximab (anti-TNFα), rituximab (anti-CD20), interferon, basiliximab (anti-IL2), or trastuzumab (anti-HER2) [20, 43]. 

Some of these reactions could be attributed to IgE-mediated mechanisms (e.g., to cetuximab); however, in many of these reactions, other mechanisms could play a role in the activation of the complement system. After administration of infliximab, most patients develop IgG antibodies (HACA) against this preparation; however, these antibodies are not necessarily clinically relevant. Late-type reactions usually occur more than 6 hours after application and could be attributable to antibodies or cellular mechanisms. T cell-mediated reactions after administration of a biologic seem to be rare. Delayed hypersensitivity reactions (> 2 hours up to several days) can occur after the use of various monoclonal antibodies, e.g., omalizumab, infliximab, trastuzumab, daclizumab, basiliximab, natalizumab, gemtuzumab, and interleukin-2 [18, 39]. The risk of developing hypersensitivity reactions is influenced by the drug’s application route (higher risk with intravenous administration compared to subcutaneous administration) and the doses of immunosuppressive comedication (e.g., methotrexate) [43].

Cetuximab [12]

Anaphylactic reactions after administration of cetuximab (chimeric IgG1-mAB against EGFR) occur in ~ 5% of patients, frequently even with the first infusion, and have a severe course in 50% of cases. In a group of 76 patients treated in Tennessee, USA, approximately 1 out of 3 developed an anaphylactic reaction. This could suggest a genetic predisposition but also particular environmental factors. Pre-existing IgE antibodies against a component of this monoclonal antibody, namely against the oligosaccharide galactose-α-1,3-galactose (α-Gal), could be demonstrated. This allergenic structure is found in the heavy chain of the Fab fragment of cetuximab. It has been speculated that sensitization against α-Gal could have taken place even before the first drug administration in many patients, e.g., via food containing animal proteins, via tick bites, or via parasite infestation (e.g., amoebas). Tools for routine determination of specific IgE against α-Gal will probably be available soon (e.g., manufactured by Phadia, Freiburg, Germany). 

Omalizumab [13]

Omalizumab has marketing authorization for the treatment of severe asthma but is also used in other indications (e.g., in urticaria or atopic eczema). The Omalizumab-Associated Anaphylaxis Joint Task Force found that anaphylactic reactions to omalizumab occur in ~ 0.09% of patients receiving this drug. 61% of reactions occurred within 2 hours after the first 3 injections, 14% within 0.5 hours after the 4th or later injections. Skin prick tests for diagnosis can probably be carried out with the pure preparation without causing irritant reactions; intradermal testing should be carried out using a dilution of 1 : 100,000 (1.2 mg/mL). The task force recommends monitoring patients for at least 2 hours after the first 3 injections and for 30 minutes after subsequent injections. Patients should be informed about possible anaphylactic symptoms, and they are even recommended to carry an autoinjector on the day of injection. 

Rituximab

Rituximab is mainly used in the treatment of lymphomas and rheumatoid arthritis; recently, there has also been increasing off-label use in inflammatory dermatoses (e.g., pustular dermatoses, dermatomyositis, atopic eczema). In ~ 18% of treated patients, adverse reactions with urticaria, fever, chills, angioedema, and drop in blood pressure occur immediately after the first infusion. However, these reactions are only rarely severe. There are no reliable data on skin testing [20]. 

**Platinum-based chemotherapeutic drugs [24, 31]**

Platinum-based chemotherapeutic drugs are frequently used in various oncologic diseases. Repeated administration is associated with an increased risk of hypersensitivity reactions (Table 1). The clinical manifestations range from purely cutaneous symptoms (pruritus, urticaria) to anaphylactic shock. Positive skin tests have been reported for carboplatin, cisplatin, and oxaliplatin; however, a high rate of false negative results has been observed. The dosages have to be chosen very carefully (e.g., skin prick test with carboplatin: 10 mg/mL; intradermal test with carboplatin: 5 mg/mL). For risk stratification, repeated skin tests seem to be useful. 

### Others 

**Proton pump inhibitors [32]**

Immediate-type reactions, some of which severe, have been reported after the use of proton pump inhibitors (e.g., omeprazole, lansoprazole, pantoprazole). Considering their wide use, these reactions seem to occur only very rarely; cross-reactions between the preparations are possible. Dilutions of 1:10 and 1:100 have been indicated to be adequate for skin testing. 

**Glucocorticosteroids [21, 42, 44]**

In addition to contact-allergic reactions after local application, glucocorticosteroids can also cause immediate-type or late-type reactions after inhalation or – rarely – after systemic application. Immunologic mechanisms for both types of reactions have been suggested in case reports. Thus, skin tests with glucocorticosteroids are also useful for the diagnostic work-up of systemic reactions. 

The following risk factors for immediate-type reactions to glucocorticosteroids have been suggested: pre-existing asthma, known intolerance to acetylsalicylic acid (ASA) / nonsteroidal anti-inflammatory drugs (NSAIDs), multiple high-dose administration of glucocorticosteroids, IV administration. The drugs that are thought to be most frequently related to adverse effects after systemic application are methylprednisolone (succinate) and hydrocortisone. Glucocorticosteroids are classified into five substance classes based on their structure, and cross-reactions between them are considered likely; this holds true for both local and systemic application (Table 2). 

**Antihistamines**

Immediate-type (e.g., urticaria due to cetirizine [9]) or late-type (e.g., maculopapular exanthema after use of cetirizine with cross-reactivity to hydroxyzine [22, 29], AGEP after use of hydroxyzine [45], fixed drug eruption after use of dimenhydrinate [38]) reactions have been reported but are very rare. For late-type reactions, skin testing (e.g., hydroxyzine 10% in petrolatum) has occasionally proven useful. 

**Insulin [17]**

Since human insulin preparations have been introduced, adverse effects have been significantly reduced. However, some cases of local reactions or systemic symptoms culminating in anaphylaxis have been reported. These are humoral- as well as cellular-mediated reactions. Delayed-type reactions can occur particularly in preparations containing zinc or protamine. Diagnostic work-up of immediate-type reactions can be carried out using skin tests and by determining specific IgE against bovine, porcine, and human insulin as well as against protamine (e.g., Phadia, Freiburg). 

**Radiocontrast agents [4, 6]**

Hypersensitivity reactions to radiocontrast agents can manifest as immediate-type and late-type reactions. Recently, it could be shown that these reactions are at least partially immunologically mediated. Indications for this are repeated reactions with re-exposure as well as positive skin and in-vitro tests (e.g., specific IgE antibodies, positive basophil activation test, positive lymphocyte transformation test). Which concentrations might be appropriate for skin testing (e.g., dilution of 1:10 for intradermal tests) is currently being studied. Whether skin tests can help to identify appropriate alternative preparations needs to be elucidated. 

**General anesthetics [28, 35, 39]**

During general anesthesia, a multitude of preparations that could lead to hypersensitivity reactions (mainly of the immediate type) are administered. This includes benzodiazepines (premedication), injectable anesthetics (e.g., thiopental, propofol, ketamine), inhalable anesthetics (e.g., isoflurane, enflurane), muscle relaxants (e.g., suxamethonium, vecuronium, alcuronium, pancuronium, atracurium), opiates (e.g., fentanyl, alfentanil), and frequently also antibiotics. 

Due to the histamine-releasing characteristics of many of these agents, skin testing is difficult, and for volatile inhalable narcotics it is virtually impossible. Real IgE-mediated reactions have, for instance, been described for thiopental and several muscle relaxants. Suxamethonium and alcuronium seem particularly frequently to be associated with adverse effects. Specific IgE against suxamethonium and also against morphine can be determined routinely (e.g., Phadia, Freiburg). Detailed recommendations for possible nonirritant doses or for other preparations for skin testing are available [28]. [Table Table3]

## Therapeutic considerations in cases of drug allergy / hypersensitivity 

As a rule, affected patients will have to avoid the allergy-inducing drug in the future. If certain drug classes cannot be avoided (e.g., radiocontrast agents or general anesthetics), alternative preparations should be used. However, the possibility of cross-reactions should be kept in mind, and premedication with antihistamines and/or glucocorticosteroids should be considered. Desensitization protocols exist for several preparations (Table 4) [8, 20, 41]. 

## Conclusion 

A multitude of drugs can induce hypersensitivity reactions. Frequently, there are no standardized procedures for diagnostic allergy work-up. Thus, allergists will have to orient themselves with the help of cases described by other authors or with general guidelines. 

**Table 1.** General recommendations for skin tests for drug allergy. From [2].

**Test**	**Preparation/concentration**	**Reading**
Patch test	Max. of 30% in petrolatum (preferred vehicle), alcohol, or aqua as the highest concentration (storage > 24 hours not recommended). Powder preparations: preferably in petrolatum (or in aqua), liquid preparations in aqua, steroid hormones in alcohol.	As in patch testing for contact allergens, final reading and evaluation after 72 hours. If necessary, additional reading after 20 minutes in immediate-type reactions.
Prick test	Ingredients and, if appropriate, additives should be tested separately. Testing with the pure agent is possible; down-titration if patient history is positive for severe reactions.	Evaluation: as with inhalant allergens Immediate (after 15 minutes): – positive if wheal > 3 mm and concomitant erythema and > negative control (NaCl) Late: palpable infiltration after 24 hours
Intradermal test	Only if prick test is negative; emergency preparedness Sterile preparation necessary Fresh preparation (< 2 h) Volume of injection 0.02 – 0.04 mL	Evaluation: no generally accepted standard Immediate (after 15 – 20 minutes): e.g., positive if wheal – increased by > 3 mm as compared to baseline – reaches 2 × the size of the control wheal – is > 8 mm or > 10 mm Late: palpable infiltration after 24 – 72 hours

**Table 2.** Classification of glucocorticosteroid substance classes with regard to possible cross-reactions when used locally or systemically, including model substance for testing. Modified from [21].

	**A**	**B**	**C**	**D1**	**D2**
**Structure**	Hydrocortisone	Triamcinolon acetonide	Betamethasone	Betamethasone diproprionate	Methylprednisolone aceponate
**Test substance**	Tixocortol	Budesonide	-	Clobetasol	Hydrocortisone-17-butyrate
Local	Hydrocortisone Hydrocortisone acetate Methylprednisolone Prednisolone Tixocortol	Amcinonide Fluocinolone Halcinonide Fluocinolone Triamcinolone acetonide/ diacetate	Dexamethasone	Clobetasol proprionate Betamethasone depropionate/valerate Fluticasone Mometasone Clobetasol	Hydrocortisone-17-butyrate Hydrocortisone valerate Hydrocortisone buteparate Hydrocortisone butyrate Prednicarbate
**Oral**	Cloprednol Methylprednisolone Prednisone Prednisolone	Budesonide Triamcinolone	Betamethasone Dexamethasone	Betamethasone	Hydrocortisone

**Table 3. Table3:** Incidence and characteristics of immediate-type reactions to platinum-based chemotherapeutic drugs. Modified from [24, 31].

**Drug**	**Incidence**	**Characteristics**
Cisplatin	5 – 20%	Within minutes during infusion Mostly during the 4th – 8th cycle Risk increased with concomitant radiation
Carboplatin	1 – 44%	Within minutes to days after infusion Very rarely during the first 5 cycles > 35% after 5th cycle
Oxaliplatin	10 – 18.9%	Within minutes or hours Mostly after 5th cycle Mostly severe anaphylactic reactions

**Table 4.** Preparations for which desensitization protocols exist. From [8, 20, 41].

**Anti-infectives**	**Use in general internal medicine**	**Oncologic/ ** **immunologic**
**Beta-lactam antibiotics ** Streptomycin Isoniazide Rifampicin Ethambutol TMP-SMX Amprenavir Darunavir Efavirenz Enfuvirtide Nelfinavir Zidovudine	Allopurinol Insulin Acetylsalicylic acid (ASA) Omeprazole	Platinum-based drugs Taxanes Doxorubicin Trastuzumab Rituximab Infliximab
